# Prenatal diagnosis of ultrasound soft markers in a single medical center of mainland China

**DOI:** 10.1186/s13039-022-00633-x

**Published:** 2023-02-10

**Authors:** Yanhong Zhou, Siqi Wu, Jin Han, Li Zhen, Xin Yang, Ru Li, Yongling Zhang, Xiangyi Jing, Fucheng Li, Huishu Liu

**Affiliations:** 1 Department of Obstetrics, First Affiliated Hospital of Jinan University, Guangzhou, China; 2 Department of Obstetrics, Guangzhou Women and Children’s Medical Center, Guangzhou Medical University, Guangzhou, China; 3 Department of Medical Genetics and Prenatal Diagnosis, Longgang District Maternity and Child Healthcare Hospital of Shenzhen City, Shenzhen, Guangdong Province China; 4 Prenatal Diagnostic Center, Guangzhou Women and Children’s Medical Center, Guangzhou Medical University, Guangzhou, China

**Keywords:** Ultrasound soft markers (USMs), Prenatal diagnosis, Absent or hypoplastic nasal bone, Karyotype, Chromosomal microarray

## Abstract

**Background:**

There are a few studies on the chromosomal aberration of Ultrasound soft markers (USMs). The aim of this study was to determine the detection rate of clinically significant chromosomal abnormalities (CSCA) in fetuses with different USMs.

**Methods:**

This study included fetuses with USMs who underwent invasive prenatal diagnosis for karyotype and/or chromosomal microarray (CMA) by categorizing into two groups: a single USM (SUSM) and multiple USMs (MUSMs).

**Results:**

Of the 358 cases with USMs, CSCA occurred in 3.09% (8/259) and 8.08% (8/99) of the SUSM and MUSM groups, respectively (*P* < 0.05). Of 16 cases identified with CSCA, theoretically 68.75% (11/16) could be detected by karyotype, while 31.25% (5/16) could be recognized only by CMA. Among CSCA cases, the most frequent USM was an absent or hypoplastic nasal bone (62.5%, 10/16). In cases with negative karyotypes and/or CMA, follow-up results were available in 307 cases, including 292 term deliveries, 6 preterm deliveries, 8 terminations of pregnancy due to USMs, and 1 still birth.

**Conclusion:**

MUSMs increased the risk of chromosomal abnormalities. An absent or hypoplastic nasal bone was the most clinically significant marker either alone or in combination with other USMs. Most of SUSM had a good prognosis.

## Introduction

The structural anomalies associated with genetic factors can be detected by prenatal ultrasound throughout pregnancy. With the advance in ultrasound devices and the improvement of sonographers’ skills, the detection rate of ultrasound anomalies, even minor structural abnormalities, has increased. Unlike structural anomalies, soft markers are often insignificant relative to outcomes, are nonspecific and frequently seen in normal fetuses, and are often transient [1]. Ultrasound soft markers (USMs) are present in 5.9–10.0% of fetuses during ultrasound in a low-risk population [2, 3]. Some USMs, such as a hypoplastic nasal bone, ventriculomegaly, increased nuchal fold thickness, or aberrant right subclavian artery, can increase the risk of Down syndrome [4].

Noninvasive prenatal testing (NIPT) has significantly contributed to prenatal aneuploidy screening recently, but it’s limited to copy number variations detection. In the last decade, chromosomal microarray analysis (CMA) has been well studied in prenatal settings, yielding a detection rate of 1.7% and 6.0% for copy number variants (CNVs) in fetuses with normal scan and structural abnormalities over karyotype [5]. Although Sagi-Dain, et al. recommended chromosomal microarray (CMA) as a first-tier test in pregnancies with a routine ultrasound or with USMs [6], there are rare correlation and evidence between CNVs and USMs. A meta-analysis study for ultrasonographic soft markers shows the yield of CMA is 0.4% (95% CI, 0.1–0.8%) over karyotyping. However, considering the procedure-related risk for amniocentesis up to 0.12% (95% CI,  − 0.05 to 0.30%) [7], no consensus has been reached regarding the best method for prenatal diagnosis of fetuses with USMs. The risk of chromosomal abnormalities differs for different markers, whether single or multiple. Invasive testing increases the anxiety of parents and the risk of miscarriage [8], so should the invasive prenatal test be recommended?

**Fig. 1:**
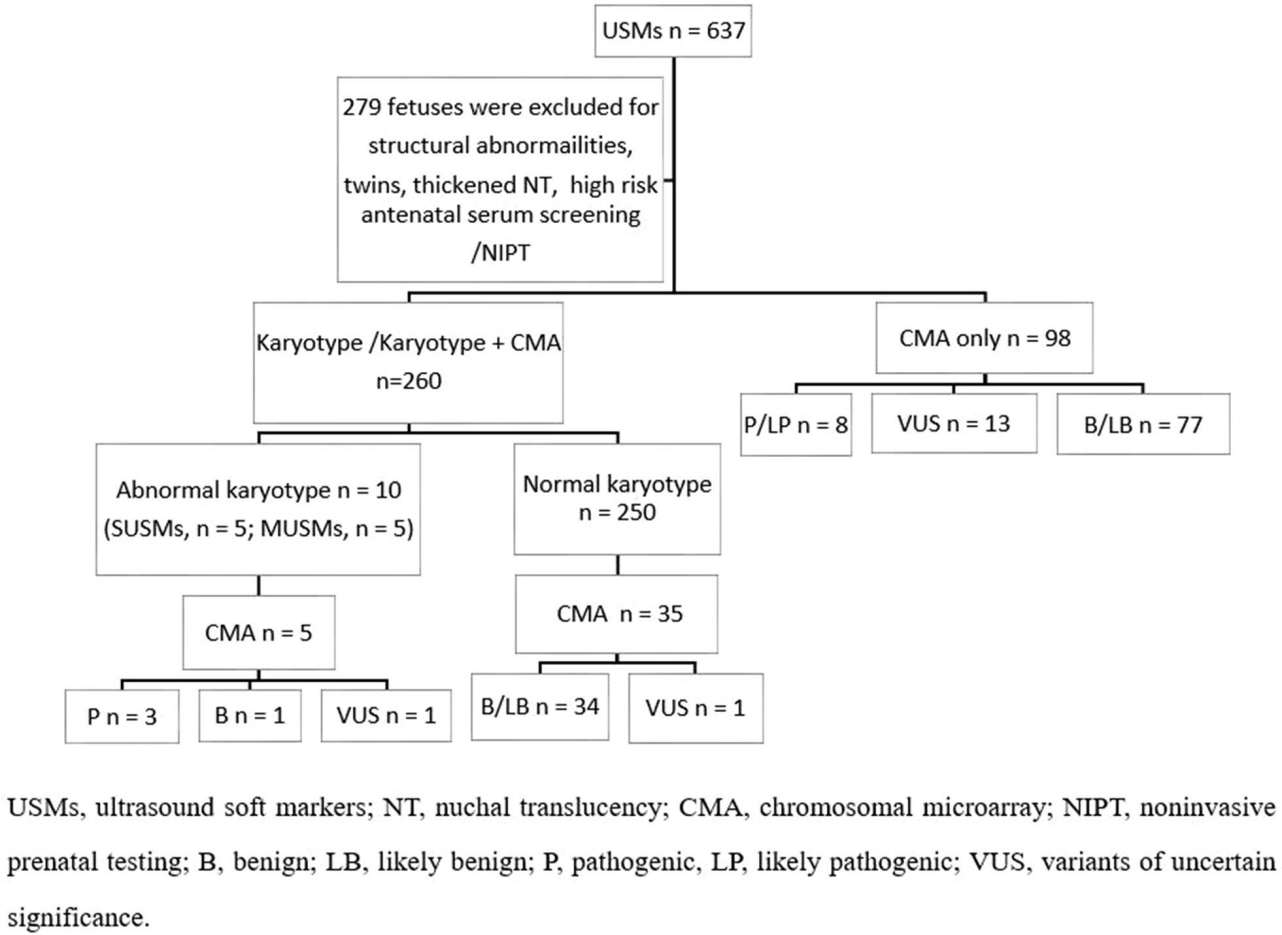
Overview of this study. *USMs* Ultrasound soft markers; *NT* Nuchal translucency; *CMA* Chromosomal microarray; *NIPT* Noninvasive prenatal testing; *B* Benign; *LB* Likely benign; *P* Pathogenic, *LP* Likely pathogenic; *VUS* Variants of uncertain significance

This retrospective study aimed to determine the detection rate of clinically significant chromosomal aberrations (CSCA) (including abnormal karyotypes and pathogenic, or likely pathogenic, copy number variations (P/LP CNVs)) in fetuses with different USMs to provide a reliable basis for clinical genetic counseling (Fig. 1).

## Materials and methods

### Study cohort

**Fig. 2:**
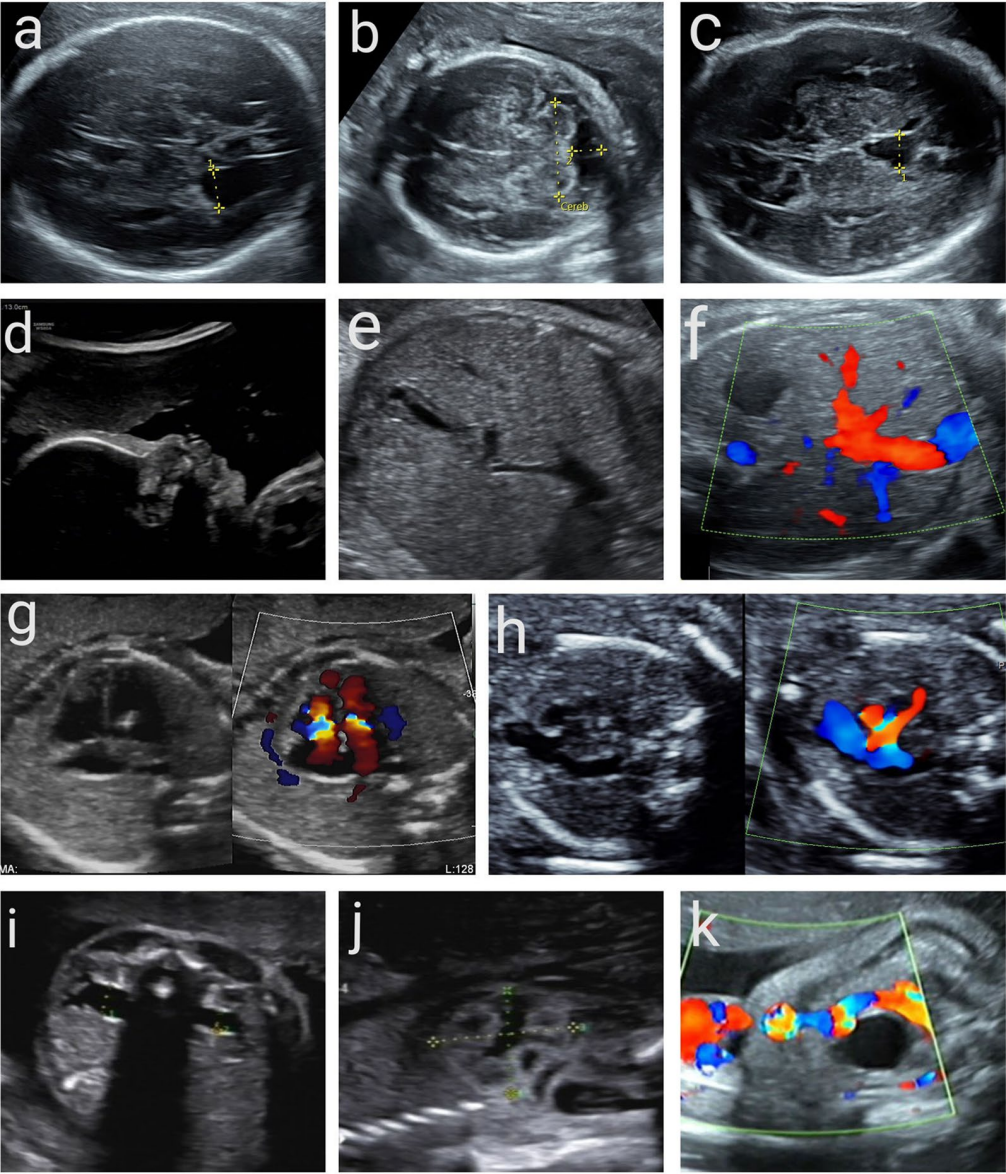
Images of USMs. **a** mild ventriculomegaly. **b** enlarged cisterna magna. **c** dilated cavum septum pellucidum. **d** absent or hypoplastic nasal bone. e. intrahepatic hyperechogenic foci. **f** persistent right umbilical vein. **g** echogenic intracardiac focus (EIF). **h** aberrant right subclavian artery. **i** mild pyelectasis. **j** hyperechogenic kidney. **k** single umbilical artery

This retrospective study included 358 fetuses with isolated USMs. They underwent invasive prenatal diagnosis at 11–38 gestational weeks for chromosomal karyotype analysis or CMA in the Prenatal Diagnosis Center of Guangzhou Women and Children’s Medical Center. Women with twins, and fetuses with nuchal translucency ≥ 3 mm, ultrasound structural abnormalities, or high risk of NIPT had been excluded. Fetuses were categorized into two groups: those with a single USM (SUSM) and those with multiple USMs (MUSMs). USMs included mild ventriculomegaly ((MV), 10–12 mm), a dilated cavum septum pellucidum (DCSP), enlarged cisterna magna (ECM), choroid plexus cysts (CPC) (uni- or bilateral), hypertelorism, absent or hypoplastic nasal bone (ANB/HNB), thickened nuchal fold (TNF), echogenic intracardiac focus (EIF), aberrant right subclavian artery (ARSA), persistent left superior vena cava (PLSVC), persistent right umbilical vein (PRUV), intra-abdominal umbilical vein stenosis (IUVS), intrahepatic hyperechogenic foci (IHF), hyperechogenic bowel (EB), hyperechogenic kidney (EK), mild pyelectasis ((MP), dilatation of the renal pelvis ≥ 4 mm in the 2nd trimester and ≥ 7 mm thereafter), shortened long bone (SLB), and single umbilical artery (SUA) (Fig. 2). Ultrasonographic markers were considered isolated when not associated with structural anomalies. The diagnosis of USMs was confirmed by two expert ultrasonographers. None of the fetuses had any structural abnormalities prior to amnio- or cordocentesis. Patients who received amniocentesis or cordocentesis gave informed consent for performing chromosomal karyotype or CMA analysis. The study protocol was approved by the Institutional Ethics Committee of Guangzhou Women and Children’s Medical Center. Clinically significant cases were recorded as chromosomal abnormalities (including aneuploidy and other abnormal karyotypes) and P/LP CNVs.

### DNA extraction and chromosomal microarray analysis

Genomic DNA was extracted from the uncultured amniotic fluid and cord blood samples using QIAamp DNA Blood Mini kits (Qiagen, Germany) and was quantified using a NanoDrop spectrophotometer. DNA quality was checked by agarose gel electrophoresis. A genome-wide, high-resolution, single nucleotide polymorphism (SNP) array, CytoScan HD (Affymetrix, Santa Clara, CA), including both SNPs and oligonucleotide probes, was used. Experimental procedures included DNA digestion, ligation, polymerase chain reaction amplification, fragmentation, labeling, and hybridization of the arrays. The reporting threshold for copy number variants (CNVs) was set at 100 kb, with a marker count of ≥ 50 kb. Results were analyzed using Chromosome Analysis Suite software and categorized based on American College of Medical Genetics and Genomics (ACMG) guidelines [9]. According to ACMG guidelines, CNVs were classified as benign, likely benign, pathogenic (P), likely pathogenic (LP), or variants of uncertain significance (VUS). Alterations coinciding with known polymorphic CNVs were interpreted as benign. In our study, P/LP CNVs were recorded as clinically significant CNVs. The following publicly available databases were used: the Database of Genomic Variants (http://projects.tcag.ca/variation/), UCSC (http://genome.ucsc.edu/), OMIM (http://www.omim.org), the DECIPHER database (https://devipher.sanger.ac.uk/), and ISCA (https://www.iscaconsortium.org/).

### Clinical follow-up assessment

Information on the delivery and status of the infant were obtained through the hospital information system. The infants’ health status was followed up for at least one year after birth. Adverse pregnancy outcomes included chromosomal defects, termination of pregnancy due to USMs, premature delivery, stillbirths, and perinatal or infant death.

### Statistical analysis

SPSS 25.0 was used for statistical analysis. Differences between the SUSM and MUSM groups were analyzed using the X2 test. A *P* value < 0.05 was identified as statistically significant.

## Results

The median age of the women in our study was 28 (19–43) years old, and the median gestational week was 26 (11–35). Of the 358 cases with USMs, 260 women elected karyotype or karyotype+CMA, while 98 chose CMA only. There were a total of 10 cases with abnormal karyotype, and 5 underwent CMA. There were 3 fetuses with pathogenic CNVs, 1 benign CNV, and 1 VUS CNV. Among the 250 cases with a normal karyotype, 34 fetuses were confirmed as benign and 1 had a VUS by CMA technique. Of the 98 CMA only cases, 8 P/LP CNVs (1 trisomy 21 and 7 P/LP CNVs), 13 VUS CNVs, and 77 benign CNVs were detected. The flow chart of this study is shown in Fig. 1.

There was a clinically significant difference in the percentage of chromosomal aberrations between the SUSM (3.09%, 8/259) and MUSM (8.08%, 8/99) groups (*P* = 0.049, Table 1). The most frequent USMs were ANB/HNB (n = 142), followed by MV (n = 63), SUA (n = 58), ECM (n = 37), CPC (n = 27), EIF (n = 27), SLB (n = 24), EB (n = 20), PLSVC (n = 19), ARSA (n = 18), PRUV (n = 13), TNF (n = 8), IHF (n = 8), MP (n = 6), EK (n = 4), DCSP (n = 4), IUVS (n = 2), and hypertelorism (n = 1). Several USMs were not detected, including DCSP, ECM, hypertelorism, TNF, IHF, and MP.

**Table 1:** Clinically significant aberrations

		Single USM	Multiple USMs	Total No. (%)
Euploid (n, %)		251 (73.39%)	91 (26.61%)	342
Total (n, %)		8 (3.09%)^A^	8 (8.08%)^B^	16 (4.47%)
Chromosomal syndrome disease (n, %)		4 (1.54%)	5 (5.05%)	9 (2.51%)
P/LP CVNs	≥ 10 Mb (n, %)	1 (0.39%)	1 (1.01%)	2 (0.56%)
	< 10 Mb (n, %)	3 (1.16%)	2 (2.02%)	5 (1.40%)
Total (n, %)		259 (72.3%)	99 (27.7%)	358

Chi-squared (X^2^) test was applied to compare chromosomal aberrations between the 2 groups, *P* = 0.049. *CVN* Copy number variants; *P/LP* Pathogenic/likely pathogenic; *USM* Ultrasound soft marker

A total of 16 cases were identified with CSCA. The karyotype can only detect chromosomal abnormalities > 10 Mb, while NIPT only detects numerical abnormalities of chromosomes 21, 18, 13, and the sex chromosomes. Of the 16 chromosomal abnormalities identified in this study, theoretically, 68.75% (11/16) could be detected by karyotype, 50% (8/16) by NIPT, and 31.25% (5/16) could only be recognized by CMA (Table 2). Most women chose to terminate these pregnancies; however, two fetuses with sex chromosome abnormalities (45,X[34]/46,XY[8] and 47,XXX[3]/46,XX[33]) were liveborn.

**Table 2:** Case summary of clinically significant chromosomal aberrations by invasive prenatal diagnosis (n = 16)

Case No.	Maternal age	Chromosomal abnormalities	USMs	Outcome
Chromosome disease				
1	33	Trisomy 18	HNB, SUA, CPC, EIF	TOP
2	32	Trisomy 21	ANB, SUA, PLSVC, ARSA	TOP
3	34	Trisomy 21	ANB, IUVS	TOP
4	36	Trisomy 21	ANB, MV	TOP
5	29	47,XXY	ANB	TOP
6	27	46,XN,der(13)	SUA, EIF, EB	TOP
7	34	45,X[34]/46,XY[8]	SUA	Liveborn
8	24	47,XXX[3]/46,XX[33]	CPC	Liveborn
9	25	46,X,i(X)(q10)[77]/45,X[16]/47,X,2i(X)(q10)[4]/46,X,del(X)(p10)[3]	MV	TOP
P/LP CNVs				
10	24	Deletion: 6p21.1-p12.3 (43,354,944–46,335,169)X1; 2.98 Mb	HNB, SLB	TOP
11	28	Deletion: 17q12 (34,822,492–36,404,104) X1; 1.58 Mb	EK, PRUV	TOP
12	27	Deletion: 18p11.32p11.31 (2,275,728–4,802,274)X1; 2.53 Mb	ANB	TOP
13	28	Deletion: Xp22.33 (1,240,318–3,185,613)X1; 1.95 Mb	HNB	TOP
14	29	Duplication: 22q11.21 (18,640,729–21,465,659)X3; 2.82 Mb	ANB	TOP
15	25	Duplication: 6p25.3p22.1 (867,006–28,132,161)X3; 27.27 Mb	HNB, SUA	TOP
16	34	Mosaicism: 12p13.33p11.1((173,786–33,865,197)X2-3; 33.69 Mb	SLB	TOP

*ANB/HNB* Absent or hypoplastic nasal bone; *ARSA* Aberrant right subclavian artery; *CNV* Copy number variations; *CPC* Choroid plexus cysts; *EB* Hyperechogenic bowel; *EIF* Echogenic intracardiac focus; *EK* Hyperechogenic kidney; *IUVS* Intra-abdominal umbilical vein stenosis; *MV* Mild ventriculomegaly; *P/LP* Pathogenic or likely pathogenic; *PLSVC* Persistent left superior vena cava; *PRUV* Persistent right umbilical vein; *SLB* Shortened long bone; *SUA* Single umbilical artery; *TOP* Termination of pregnancy

In the SUSM group, 8 fetuses were found to have clinically significant chromosomal aberrations. Fifty percent of the cases (4/8) had an ANB/HNB, which included 1 case of 47, XXY, and 3 cases of pathogenic CNVs < 10 Mb. The remaining 4 cases had an SUA, CPCs, MV, or SLB. Three of these cases had sex chromosome mosaicism and one case had CNV > 10 Mb.

In the MUSMs group, 8 fetuses had clinically significant chromosomal aberrations. There were 5 (62.5%) cases of recognized chromosomal syndromes, 2 (25%) cases were P/LP CNVs < 10 Mb, and 1 (12.5%) case of pathogenic CNV > 10 Mb. In this group, the most frequent soft markers were ANB/HNB (6/8, 75%), SUA (4/8, 50%), and EIF (2/8, 25%).

In cases with negative karyotypes and/or CMA, follow-up results were available in 307 cases, including 292 term deliveries, 6 preterm deliveries, 8 terminations of pregnancy due to USMs, and 1 stillbirth. A case of a minor ventricular septal defect was detected after birth, but prenatal ultrasound in that case only demonstrated an SUA. None of the eight pregnancies terminated due to USMs were performed at our center. One fetus with isolated EB was confirmed to have an intestinal obstruction following delivery. In general, after excluding chromosomal abnormalities, fetuses with USMs had a good prognosis.

## Discussion

This study found a statistically significant difference in the presence of CSCA between the SUSM and MUSMs groups. Wang et al. suggested an association between pathogenic copy number variations (pCNVs) and fetal with multiple USMs increasing the risk of fetal segmental aneuploidies [10]. Similar to the previous study, MUSMs increased the risk of chromosomal abnormalities, including known chromosomal syndromes and copy number variations. However, it is noteworthy that, chromosomal syndrome accounted for 62.5% of chromosomal aberration in MUSMs groups in our data, but the most common chromosomal aberration in MUSMs groups was pathogenic CNVs in Wang’s research. The difference may be related to selection bias. In brief, the invasive prenatal diagnosis should be recommended for fetuses with MUSMs, and a diagnostic yield of 8.08% warrants the application of CMA in pregnancies with MUSMs.

An ANB/HNB was the most common ultrasound soft marker in our study, and was the most clinically significant marker, either alone or in combination with other USMs. The detection rate of CSCA in cases with ANB/HNB was 7.04% (10/142); however, it occurred in 62.5% (10/16) of positive cases. Of note, three cases who had ANB/HNB were identified with trisomy 21. Similar to previous studies, the ANB/HNB are strong indicators for fetal Down’s syndrome [4, 11]. In addition, the risk of chromosomal defects when an ANB/HNB was associated with other soft markers (6/26, 23.08%) was much greater than for ANB/HNB (4/116, 3.45%) alone. An ANB in association with other structural abnormalities has been shown to have a higher rate of abnormal karyotypes than an isolated ANB [11], Five cases with pathogenic CNVs were observed with ANB/HNB in our data. Additionally, pathogenic CNVs were identified in 5.1–6.4% of hypoplastic nasal bone in combination with another soft marker or a structural abnormality [12, 13]. Thus, a detailed sonographic examination for fetal structure is recommended in these cases. These conclusions are also consistent with previous studies [14].

Three fetuses with a single USM consisting of SUA, CPC, or MV were detected and had mosaic aneuploidy of chromosome X. Two were live births, and the pregnancy with MVM was terminated. Several meta-analyses have shown that the presence of an SUA increased the risk of perinatal complications such as small for gestational age, oligohydramnios, polyhydramnios, gestational diabetes mellitus, and perinatal mortality, but there was no evidence that fetuses with only an SUA have an increased risk for aneuploidy [15–19]. Thus, according to the recommended management for isolated SUA, negative noninvasive prenatal testing or serum screen is sufficient for aneuploidy evaluation in pregnancies. But in the case of fetuses with isolated SUA in the third trimester, ultrasonography examination for growth assessment should not be overlooked [20]. In general, 30–50% of fetuses with trisomy 18 have a CPC, but 1–2% of healthy second trimester fetuses also have these cysts, which usually resolve by approximately 24 gestational weeks. If only a CPC is present, it is considered a normal variant. However, if a CPC is associated with other anomalies, the fetus was more likely to have trisomy 18. If CPCs are found, a detailed ultrasonographic structural screening of the fetus should be performed with particular attention to the heart and hands [21, 22].

The favorable outcome could be assured in isolated mild ventriculomegaly with 10–12 mm [23]. All of 62 fetuses with MV in our series, except for the one case of pathogenic CNV that pregnancy termination was chosen, had good neonatal outcomes. However, a prospective study suggested that the prognosis of MV was not favorable [24]. The difference in chromosomal burden and outcomes was attributed to different inclusion criteria that they defined MV as lateral ventricle width between 10 and 15 mm. Indeed, there was a higher risk of associated CNS abnormalities in fetuses with moderate (13-15 mm) than in those with mild VM (10–12 mm) [25]. Moreover, 4.3% of fetuses with mild VM and 20% with moderate VM by ultrasound were diagnosed with additional central nervous system abnormalities by prenatal magnetic resonance imaging (MRI) [26]. In short, pathogenic CNVs may be involved in the process of fetal MV and postnatal neurodevelopmental disorders, so it is necessary to perform a CMA and detailed ultrasonographic screening and MRI neurosonography [27–31]. This study supported that isolated MV without structural or chromosomal abnormalities had a good prognosis.

Of the 24 fetuses with SLB, 8.33% (n = 2) had pCNVs, one with a 6p21.1 microdeletion and one with 12p13.33 microduplication mosaicism. Some parents chose skeletal gene testing directly. Liu et al. [32] found that CMA resulted in a greater detection rate than karyotyping in fetuses with other abnormalities or a femur length (FL) 2–4 SDs below the gestational age mean during the second trimester. Gene sequencing detected clinically notable mutations in fetuses with SLB, especially those with FLs > 4 SDs below the mean. An isolated SLB, without any structural or chromosomal abnormalities, was significantly associated with intrauterine growth retardation or being small for gestational age and a poor perinatal outcome [33, 34]. Therefore, invasive prenatal diagnosis, detailed biometrical monitoring and comprehensive counseling on prognosis should be carried out for fetuses with SLBs.

In this study, no chromosomal abnormalities were found in fetuses with a single soft marker of EIF. Hu et al. found that the normal infant rate was above 95% in fetuses with EICF [24]. These suggest that isolated EIF does not increase the risk of chromosomal abnormalities, which is consistent with the recommended management for EIF from the Society for Maternal–Fetal Medicine [20]. However, the risk of chromosomal abnormalities was increased when other soft markers were present. A higher rate of fetal malformation will be found when EIFs are located in the right or both ventricles [35, 36]. Of the four fetuses with EK, one who also had a PRUV was determined to have 17q12 microdeletion syndrome. Jing et al. [37] found a strikingly high correlation between unilateral or bilateral EKs and 17q12 deletion. Therefore, prenatal testing with CMA should be offered in these cases. Chromosomal anomalies occurred in 3.3% of fetuses with isolated EB, primarily trisomy 21, and aneuploidies involving the sex chromosomes. However, isolated EB does not carry an increased risk for abnormal CMA. Fetuses with EB are at increased risk of adverse perinatal outcomes, highlighting the need for comprehensive antenatal management and postnatal follow-up [38, 39].

Approximately 90% of fetuses with a trisomy 18, and 40% of those with trisomy 21 or 13, had a CSP width > 95th percentile compared to euploid fetuses, so a large CSP should prompt a detailed ultrasound examination further to assess the risk for chromosomal abnormalities [40]. Chaoui et al. [41] noted that a dilated CSP might be a crucial sonographic marker for the presence of 22q11 deletion syndrome along with conotruncal malformations and thymic hypoplasia. However, an abnormal prenatal CSP, without an associated fetal abnormality such as aneuploidy, appears to have an expected outcome [42]. Therefore, a detailed fetal structural examination is necessary, when a DCSP is found during prenatal ultrasound screening, as an isolated DCSP does not increase the risk of chromosomal abnormalities. Prior studies have found the pretty low risk of chromosomal anomalies in fetuses with isolated ECM, and their neurodevelopmental outcome is generally favorable [43–45]. In this study there was only one fetus with hypertelorism, and they had normal chromosomes; therefore, it is limited to illustrate the significance in prenatal settings. Hypertelorism is usually associated with a syndrome but can also be isolated or caused by mass effect; therefore diagnostic testing with karyotype analysis, CMA, or gene panel or exome testing should be considered [46]. When TNF was detected, alone or in association with other soft markers, the risk for chromosomal anomalies was significantly lower than if structural malformations existed [47]. Simchen et al. found that of 21 patients with isolated IHF, one fetus had experienced a parvovirus B19 infection and one infant had trisomy 21. The remainder of the infants had a good outcome, indicating that isolated IHF had a good outcome after excluding aneuploidy and intrauterine infection [48]. In the absence of other structural anomalies or soft markers or risk factors for aneuploidy, amniocentesis for isolated MP does not appear to be warranted. However, approximately one-quarter to one-third of fetuses show a progression of pyelectasis, and a third-trimester ultrasound is recommended to identify worsening or persistent cases. Persistent or progressive pyelectasis requires postnatal evaluation or surveillance [49].

VUS has been a challenge for CMA testing, because there is insufficient evidence to determine their definitive clinical significance, and the CNV may not meet the reporting criteria established by the laboratory. In clinical practice, VUS increases the difficulty of genetic counseling and causes patient anxiety. In these cases, it is necessary to add CMA examination, detailed ultrasonography, and postpartum follow-up.

## Conclusions

MUSMs increased the risk for chromosomal abnormalities, so invasive prenatal diagnosis and CMA should be recommended in fetuses with MUSMs. An ANB/HNB was the most clinically significant marker either in isolation or in association with other USMs. Most isolated USMs were associated with a good prognosis. When USMs are found, a detailed ultrasonic examination is necessary.

## Data Availability

The original contributions presented in the study are included in the article, further inquiries can be directed to the corresponding author.
